# The Alderney Race: general hydrodynamic and particular features

**DOI:** 10.1098/rsta.2019.0492

**Published:** 2020-08-21

**Authors:** Pascal Bailly du Bois, Franck Dumas, Mehdi Morillon, Lucille Furgerot, Claire Voiseux, Emmanuel Poizot, Yann Méar, Anne-Claire Bennis

**Affiliations:** ^1^ Laboratoire de Radioécologie de Cherbourg, IRSN-LRC, Rue Max Pol Fouchet B.P. 10, 50130 Cherbourg en Cotentin, France; ^2^ Service hydrographique et océanographique de la marine – Shom, Brest, France; ^3^ LUSAC, Laboratoire Universitaire des Sciences Appliquées de Cherbourg, University Normandie, Cherbourg-en-Cotentin, France; ^4^ Conservatoire National des Arts et Métiers, Intechmer, Cherbourg-en-Cotentin, France; ^5^ Normandie Univ., UNICAEN, CNRS, UNIROUEN, Morphodynamique Continentale et Côtière (M2C), Caen, France

**Keywords:** Alderney Race, La Hague Cape, current, tide, hydrodynamics, model

## Abstract

This study presents an overview of the main hydrodynamic features of the Alderney Race strait based on *in situ* measurements and two-dimensional hydrodynamic model simulations. The strait encompasses a large amplitude of tidal properties (tidal range and tidal wave propagation) and particularly strong currents exceeding 5 m s^−1^ with associated counter currents and gyres. Variations in depth, sea bottom roughness, coastal topography and current orientation around the La Hague Cape provide access to a large variety of original hydrodynamic regimes. Some are revealed as locations with a 0.4 m drop in the mean sea level associated with strong average currents. A resonance effect associated with the offshore currents can also be observed close to the coasts. The ‘St Martin whistle’ occurs in a bay whose gyre centre oscillates with a reversal of the measured current every 5–7 min. The Alderney Race represents a particular area of interest for coastal hydrodynamic studies. The available *in situ* measurement datasets are rich with recordings of: sea levels; acoustic Doppler current profiler current profiles; surface radar currents; waves; dye experiments; surface and in-depth dissolved tracer surveys. Combined with hydrodynamic models, the complexity of this area can be further understood and knowledge of the hydrodynamic process and forcing parameters can be refined, which can be applied to other coastal areas.

This article is part of the theme issue ‘New insights on tidal dynamics and tidal energy harvesting in the Alderney Race’.

## 1. Introduction

**Figure 1.**
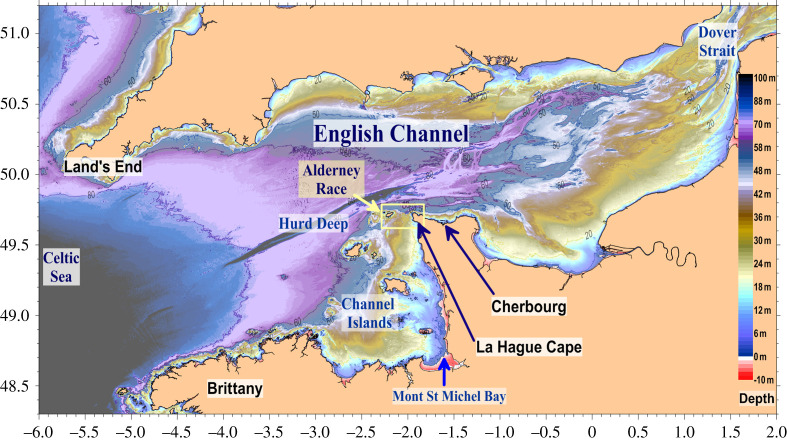
Bathymetry map of the English Channel with the main locations cited. The yellow rectangle shows the study area (figure 2). (Online version in colour.)

**Figure 2.**
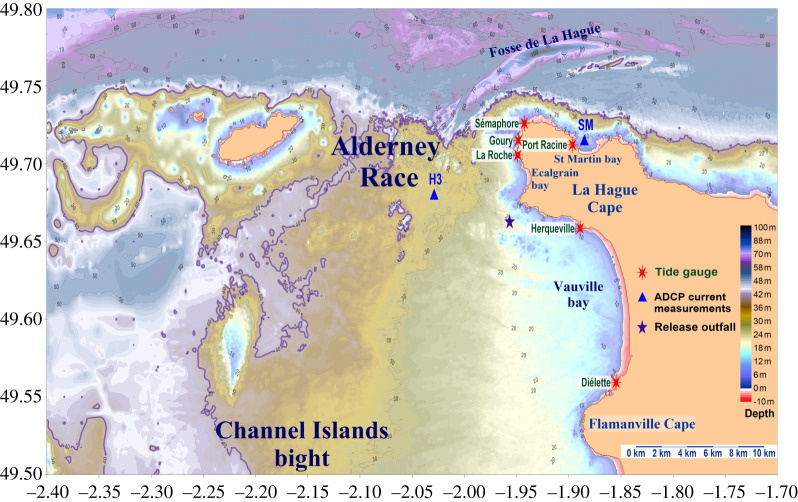
Bathymetry map of the Alderney Race and measurement locations. (Online version in colour.)

The Alderney Race is located between the La Hague Cape and the Alderney Island at the centre of the English Channel, which is part of the western European continental shelf. The English Channel (figure 1) extends longitudinally and is connected to the Atlantic Ocean to the west via the Celtic Sea between northern Brittany and Lands End (177 km in width). Seawater flows eastward into the North Sea through the Dover strait (32 km in width). Depths do not exceed 200 m and decrease westward to eastward with depths lower than 50 m in the eastern English Channel and lower than 30 m in the Channel Islands area. Two narrow depressions are located in the centre of the English Channel, north-west and north of the La Hague Cape. They are mainly oriented west to east with maximum depths of 160 and 110 m (‘Hurd Deep’ and ‘Fosse de La Hague’ also called ‘La Hague deep’, respectively). The Alderney Race shows a 30 m deep rocky platform south of 49.71° N, with a marked step in the middle of the strait. This geological fault extends 4–8 km west of Goury with a step ranging from 30 to 60 m in depth (figure 2, 40 m isobath in bold). The coasts are mostly rocky with coarse and sandy mobile sediments in the bays (Ecalgrain, St Martin) and locally finer sediments deposits in the Goury harbour. Details of the Alderney Race morphological structures are given in [1].

The general circulation of the English Channel flows eastward where it enters into the North Sea through the Dover strait. The tidal waves follow the same orientation from the west to the east with a delay of roughly 7 h (between the western entrance at the longitude of Brest and the eastern boundary at Calais): they are compatible with the propagation characteristics of a progressive long wave.

The Alderney Race is located north-west of the Cotentin peninsula, which can be seen at the tidal wave scale as a major obstacle significantly reducing the north-south section of the Channel into which the tidal waves propagate. Moreover, the Alderney Race is located on the south side of the English Channel where the tidal range is larger than the north side. Both these points schematically explain the intense hydrodynamics of this area.

This study aims to present the main hydrodynamic processes of the Alderney Race based on bathymetry, sea-level variations, current, dispersion observations and hydrodynamic model simulations. This inventory accounts for the interest of the Alderney Race as a natural laboratory for coastal hydrodynamic studies.

The introduction presents a short description of the situation and an overview of previous studies concerning the Alderney Race. The next section describes the methods applied to obtain *in situ* measurements and simulations. The results section presents the particular features observed concerning the tide and associated sea level and currents. The discussion section includes possible ways to improve the understanding of the hydrodynamics in energetic regimes and how the Alderney Race may be valuable as a toolbox to study extreme coastal hydrodynamic processes. Lastly, examples of research perspectives are presented.

The conclusion syntheses the main results obtained and the value of the Alderney Race in improving knowledge of hydrodynamic processes and forcing parameters that could be applied in other coastal areas.

### (a) Scale model

Before the nuclear reprocessing plant at La Hague was set up, a scale model was built by the Institut de Mécanique de Grenoble (currently the Coriolis platform operated at UMR n°5519 CNRS-LEGI) in a rotating plate representative of the Channel. It has been applied in the investigation of the currents around the La Hague Cape and the study of dispersion of dissolved releases (dye experiments [2]). It has also been applied in the study of the tidal propagation at larger scale in the English Channel [3].

### (b) Dye experiments

Several dye experiments were performed in 1962 and 1963 before the nuclear reprocessing plant at La Hague was set up using rhodamine *β* at the planned outfall for radioactive releases. These experiments were designed to catch the dispersion of the dissolved releases over days to weeks following the releases [4]. Recent dye experiments were conducted south of the La Hague Cape (DISCO project) and are still underway.

### (c) Radiotracer dispersion

The Orano nuclear reprocessing plant at La Hague has performed controlled releases of dissolved radionuclides since 1963 in the Alderney Race (a black star shows the location of the release outfall in figure 2). Many studies have been performed to investigate the behaviour of these dissolved radionuclide releases in the past 30 years. They involve several time and space scales, ranging from minutes and kilometres following an individual release [5,6] and up to years and thousands of kilometres [7–10]. As the dispersion of dissolved releases is governed by hydrodynamics, the measurements of radionuclide concentrations and plume dispersion reveal the cumulated effects of the current and diffusion effects at different scales.

The methods applied to sample and measure the dissolved radioactivity are described in [5,10]. At short scales, tritium associated with seawater (HTO) is the main radiotracer measured and 10 ml seawater samples were sufficient to determine the tritium concentrations. More than 22 000 measurements are given, available in [11]. These measurements enable horizontal and vertical maps of the plumes of individual releases to be plotted of dispersion around the La Hague Cape.

### (d) Drifters

Drifters were deployed in 1962 and 1963 during pre-operation studies of the La Hague reprocessing plant [4]. In March 2007 two ARGOS drifters were released [5]; these drifters are composed of a surface buoy with a GPS receiver and a transmission link with the ARGOS constellation. The buoys are linked with a cable to a holey sock that is weighted at its base and immersed 15 m below the surface. Their drift is representative of currents in this layer. These two drifters were released at a distance of 100 m apart at the same time in the Alderney Race and then tracked every 30 min over 13 days. They were separated by a distance of 54 km from each other at the time of retrieval [5].

## 2. Methods

### (a) Bathymetry

Precise knowledge of the bathymetry is a prerequisite for most marine hydrodynamic studies. This is particularly the case in the Alderney Race as the bathymetry determines the frame of the ‘pipe’ where the seawater flows. It explains the current variations at short and large scales. Most bathymetric data are provided by the French hydrographic and oceanographic service (Shom) [12]. We supplemented these data with measurements obtained during campaigns on board the Genavir Haliotis vessel. A compilation of all available measurements from Shom and IRSN is provided in [1]. As hydrodynamic models request homogeneous datasets from the different sources available, a method was designed to build such a dataset [13]. This method allows bathymetric maps suitable for hydrodynamic models to be calculated with a horizontal resolution from 1 m up to 10 km.

### (b) Sea-level variations

Sea-level fluctuations have been recorded over many years by Shom to investigate the tidal characteristics and mean sea-level variations for hydrographic purposes (navigation maps referenced to a safe vertical level). These measurements are almost continuous for the main harbours and during restricted periods for the smallest harbours. Maximum tidal ranges exhibit large variations from the north to the south of the La Hague Cape (figure 2). As a frontier between the eastern English Channel and the Channel Islands area, this zone has variable responses to the different forcing such as wind, atmospheric pressure, bottom and surface stress in the water column. From this perspective, short period tide gauge measurements have been performed to improve the description of the tidal propagation around the La Hague Cape and to better calibrate the hydrodynamic models.

As the coastal zone area is made of rocks, pebbles and sand without easy access to infrastructure required to perform classical tide gauge measurements, we developed a method to obtain sea-level measurements without designated infrastructures. This method is based on pressure gauges provided by the company NKE^©^ that enables autonomous measurements during long periods (years) at high frequency (1 Hz) to be taken. These light instruments (2.8 × 11.4 cm, 85 g) are fixed inside cinderblocks filled with 5–10 kg of lead to ensure high stability during energetic sea states and not recovering by other divers. They were placed on the sea floor during low tide, 1–3 m below the lower sea level. These tide gauges were retrieved and re-deployed after 2–12 months of continuous recording with checking of the pressure and clock drift at each recovery. The typical configuration was measurement of the seawater pressure and temperature at 1 Hz. These parameters were then averaged every 30 s before recording. This setting enabled most of the wave influence to be filtered and long-term continuous records to be stored in the instruments' internal memory. The tide gauge locations are represented by red stars in figure 2.

Initial measurements (2006–2010) were performed without terrestrial reference. A system to obtain such a reference was applied from 2010 to 2013. It uses a GPS-RTK system to record the precise altitude of a nail fixed into a rock as close as possible to the tide gauge location. This system provides the altitude measurement referenced to the geoid WGS84 with a vertical precision lower than 1 cm.

**Figure 3.**
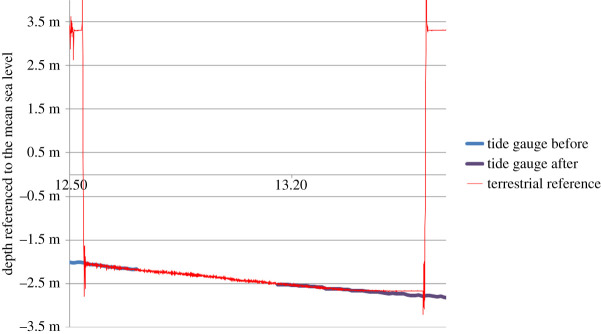
Example of sea-level variations measured by the bottom tide gauge before (blue) and after (purple) recovery, and by the terrestrial referenced pressure gauge (red). Level 0 corresponds to the measured mean sea level. The terrestrial reference is applied to measure the vertical position of the tide gauges by curve superposition. (Online version in colour.)

To obtain the vertical difference between the submersed tide gauge and the terrestrial reference, we measured the sea-level altitude with a pressure gauge deployed at the sea surface on a floating device. This gauge was connected by a 20 m tube filled with water to a flexible bag full of sweet water placed at the location of the terrestrial reference nail. This system continuously recorded the hydrostatic pressure that corresponds to the altitude of the sea level referenced to the altitude of the terrestrial reference. We measured the sea-level fluctuations using this device during each retrieval and deployment process from 2010 to 2013. Through adjustment with the measurements obtained from the gauges recording the sea-level altitude from the bottom, the vertical position of the bottom tide gauges can be deduced with a precision better than 5 cm (figure 3). All the records were checked to confirm the vertical reference at the beginning and end of the deployments. Corrections where made when changes occurred, which sometimes happened during storm events where abrupt changes were observed (probably when the gauge fell into a hole). Slow drifts of the pressure gauges were also observed (a few centimetres). They were corrected by linear interpolation between the beginning and end of the deployment. Corrections of the atmospheric pressure changes during all the deployment periods were also performed.

### (c) Instantaneous current measurements

Current profiles have been recorded at different locations since 2003 by bottom-mounted acoustic Doppler current profilers (1 MHz ADCP) with measurement durations of 7–13 h. During the HYD2M project (2017–2018), three 500 kHz ADCP were deployed in the Alderney Race with high frequency measurement (1–2 Hz) for up to 1 year. These last measurements provide detailed information concerning current variability, wave effects and fluctuations that are described elsewhere [14,15] and in this theme issue [16]. In this study, we only used the average current through the water column at one station for comparison with two-dimensional hydrodynamic model results. ADCP locations used in this study are indicated with blue triangles in figure 2.

### (d) Numerical hydrodynamic models

The efficiency of numerical hydrodynamic models has been proven in the English Channel, particularly to simulate the behaviour of dissolved radionuclide releases at various time scales (from hours to years) from the nuclear reprocessing plant at La Hague [5,10,11,17–20].

The models applied here are based on the model for applications at regional scale (MARS) developed by the French Ifremer Institute since 1987 [21]. The framework of this model relies on various assumptions: use of the ‘Lagrangian barycentric’ method in two dimensions to filter out the tidal signal; application of the non-stationary Saint-Venant equations (i.e. two-dimensional horizontal); and the three-dimensional primitive equations. The MARS model is described in detail in [21], it used finite difference discretization within the framework of Boussinesq approximation and hydrostaticity. A uniform horizontal ‘Arakawa-C’ discretization [22] was used, the model gets a wetting and drying capability.

Modelling studies [5,9,23] have demonstrated that models using two-dimensional horizontal approximation (i.e. shallow-water equations) can quite accurately simulate the main currents and dissolved-substance concentrations in non-stratified areas mostly driven by tidal and meteorological forcings. The English Channel, east of 3°W, is seldom thermally stratified or stratified in terms of salinity; this is due to the strong tidally induced mixing, particularly in the Alderney Race. Baroclinic processes are thought to play a significant role in the propagation of the barotropic tide (due to energy dissipation within the internal tide processes) but this was empirically corrected within the scope of this study by adjusting the bottom friction at large scales.

The hydrodynamic model applied here is principally the two-dimensional version of MARS, which is sufficient to appraise the sea-level variations and depth-averaged currents in the Alderney Race. More detailed studies will be presented in this issue by using three-dimensional models that reproduce the current evolution from the surface to the bottom.

**Figure 4.**
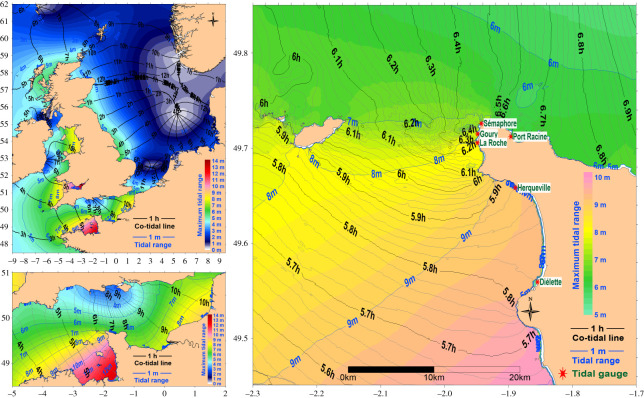
Co-tidal lines in hours (black lines). Background: maximum tidal range in metres (blue lines). (Online version in colour.)

**Figure 5.**
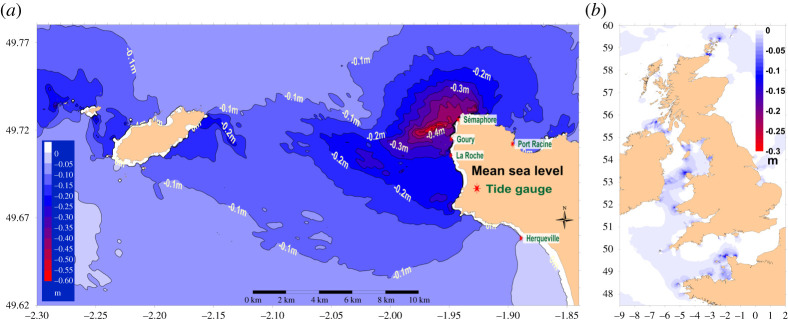
Mean sea levels calculated between 1 March 2015 and 29 April 2015 (two moon cycles). (*a*) Alderney race, (*b*) European continental shelf. (Online version in colour.)

The model involves a nesting strategy, starting from a broad region covering the entire North-West European continental shelf (with a 5.6 km grid resolution) down to a detailed domain covering a few tens of kilometres. The tidal spectra at the open boundaries of the model have the 14 most significant components of the tidal atlas FES2014 (updated from [24]). Data exchange between domains is unidirectional (one-way offline nesting), from the broadest coverage model to the finest-scale high-resolution model. Each rank of the nested models provides the open boundary conditions to the next embedded rank. The bathymetry at the grid nodes of the different models is estimated with the method described in [13] from various data sources [5,13]. We applied models covering figures 1, 2, 4 and 5 with horizontal resolutions of 2000, 1500, 400 and 110 m. For the comparison with the tide gauge measurements, the simulations were run during the whole measurement period (8.9 years) with the 400 m model. The spatial high-resolution tidal characteristics (mean sea level, largest tidal range, average and maximum currents) were computed over two tidal cycles with the 110 m model (1 March 2015 to 29 April 2015). This period encompassed a wide range of tidal conditions (from neap to spring) including the largest tides in the last 10 years (2010–2020). This period with low and high tide was taken as representative of the possible tidal characteristics. Specific simulations were performed for some corresponding measurement periods. We calculated co-tidal charts by simulating a real situation accounting for the 14 main tidal harmonics and for a mean tide (3 March 2015). To obtain better precision for the tide propagation, we recorded the time when, after full tide, the sea level crossed the mean sea level obtained during the whole tidal cycle. The results were obtained within hours after the high tide at the off Brest Shom reference location for tide propagation modulo for the tide period (12.42 h).

## 3. Results

### (a) Tide

Figure 4 shows the tidal wave propagation from the continental to the local scale. It accounts for a real situation with the different tidal harmonics, but nevertheless it is quite similar to the already published M2 tide co-tidal charts [25–29]. The tidal wave is essentially generated in the North Atlantic water masses. It is first amplified when it crosses the continental shelf break at the entrance of the Celtic Sea and all along its propagation from west to east over the European continental shelf. The orientation and morphology of the English Channel causes additional amplification of the tidal amplitude in the Normand-Breton Gulf (or the British Isles area), principally in the Mont St Michel Bay (tidal ranges here reach 14 m). The Channel Islands area represents a dead end where the tidal wave is magnified by the coastal topography, which explains the maximum tidal range observed (figure 4). After filling and emptying the British Isles area during the flood and ebb phases of the tidal propagation, the tidal wave passes through a narrowing in the middle of the Channel west of the La Hague Cape. The water body in the British Isles Gulf moves back and forth at the tidal period (i.e. with the period of the main M2 tidal component, 12.42 h) and thus travels highly constrained to the east through the bottleneck of the Alderney race.

From the measurements performed around the La Hague Cape between 2005 and 2013, it is possible to assess the tidal wave propagation from south to north of the La Hague Cape. Average results are presented in table 1. The tidal wave is almost synchronic from the north to the south of the Vauville bay (12 km length, 2 min 7 s delay for high tide): the largest tidal range was observed in the north (Herqueville). The co-tidal lines computed from the numerical model show a divergence in the Vauville Bay with a corresponding delay of 3.36 min (figure 4) with a smaller tidal range at Dielette than the one measured at Herqueville. To the north of the Vauville bay the tide propagates as a progressive Kelvin wave (from south to the north) that follows the Cape coastline. The tidal boundary represented by the La Hague Cape is illustrated by the tidal slow-down propagation measured between the south—Herqueville and the north of the Cape—Port Racine (in the St Martin Bay, 5.3 km distance in a straight line), associated with a convergence of co-tidal lines (figure 4). With a mean tidal range of 6.15 m at Herqueville and 3.88 m at Port Racine, the mean tidal range difference is 2.27 m with a mean delay of 45 min for the high tide. For the highest measured tidal range the difference is 4.15 m.

**Table 1.** The measured and simulated tide propagation from the south to the north of the La Hague Cape: tidal range, mean propagation (high tide) and mean sea levels. Delays, mean sea-level variations with space (D*Hm*/D*x*) and alongshore apparent velocity of the high tide propagation (D*x*/D*t*) are referenced to the previous column/location. The simulation covers 8.9 years, the whole measurement period, with a model having a 400 m horizontal mesh size.

		Diélette	Herqueville	Auderville La Roche	Auderville Goury	Auderville semaphore	Port Racine (St Martin Bay)	Cherbourg
measurement duration (years) model: 8.9 years		0.9 y	6.6 y	2.9 y	5.2 y	0.4 y	4.4 y	7.8 y
tidal range								
measurements	minimum	1.37 m	1.56 m	1.73 m	1.06 m	1.61 m	0.95 m	1.01 m
	mean (Hm)	5.76 m	6.15 m	5.36 m	4.92 m	4.33 m	3.88 m	3.94 m
	maximum	9.78 m	10.62 m	8.96 m	8.65 m	7.24 m	6.47 m	6.68 m
model	minimum	0.77 m	0.74 m	0.90 m	0.82 m	0.79 m	0.63 m	0.66 m
	mean (Hm)	5.97 m	5.85 m	5.21 m	5.00 m	4.56 m	4.07 m	3.93 m
	maximum	9.39 m	9.21 m	8.19 m	7.92 m	7.24 m	6.37 m	6.18 m
tidal wave propagation from Diélette to Cherbourg								
distance from Diélette (D*x*)		0.00 km	12.03 km	7.04 km	0.74 km	1.37 km	4.01 km	19.92 km
measurements	D*t* (h:mm)	0:00	0:02	0:11	0:17	0:35	0:47	1:13
	D*Hm*/D*x* (cm km^−1^)		3.2 cm	−11.2 cm	−58.8 cm	−43.2 cm	−11.3 cm	0.3 cm
	D*x*/D*t* (km h^−1^)		341 km h^−1^	44 km h^−1^	7 km h^−1^	5 km h^−1^	20 km h^−1^	46 km h^−1^
model	D*t* (h:mm)	0:00	0:04	0:13	0:18	0:39	0:50	1:19
	D*Hm*/D*x* (cm km^−1^)		−1.0 cm	−9.1 cm	−27.1 cm	−32.7 cm	−12.2 cm	−0.7 cm
	D*x*/D*t* (km h^−1^)		158 km h^−1^	49 km h^−1^	9 km h^−1^	4 km h^−1^	21 km h^−1^	41 km h^−1^
mean sea level (ref IGN69)								
measurements			0.56 m	0.13 m	0.39 m	0.34 m	0.53 m	
Shom RAF2017		0.54 m			0.23 m			0.59 m
model Mars2D		0.62 m	0.63 m	0.43 m	0.35 m	0.20 m	0.54 m	0.59 m

The tide gauges at different locations enabled the tide propagation around the cape to be assessed. The average tidal range variation with distance (D*Hm*/D*x* in table 1) shows how the mean tidal range varies according to the location with values reaching 0.43–0.58 m km^−1^ between La Roche and the semaphore. The velocity of the high tide propagation is correlated to this evolution with the lowest speed in the same area (D*x*/D*t* = 5–7 km h^−1^). For comparison the average velocity of the tidal wave propagation in the English Channel was calculated as 73 km h^−1^, between the west Brittany and the Dover Strait ([28] 79 km h^−1^ for the M2 tide around the UK coastline).

Figure 4 shows the simulated maximum tidal range. The discrepancies between simulated and measured mean tides are within 1.7–5.3%, and the maximum variation relates to the Herqueville station with the measured maximum tide 13% higher than the simulated one (10.62 versus 9.21 m). If the average tidal range is close to the measured one (deviation between −5% and 3%), the minimum tidal ranges are underestimated by the model (differences between 22% and 53%). This could be mostly explained by the sea-level variations associated with meteorological events (atmospheric pressure, wind forcing) that have a higher relative influence during low tides. The maximum tidal ranges were better simulated (differences between 0% and 13%). The tide propagation was similar to measured and simulated with differences in time arrival below 6 min. The measured and simulated tidal range variation shows differences between Goury and the semaphore where the tidal gradient is more important. The relative errors in the model were largest at Herqueville with underestimation of the tidal range.

Another way to estimate the influence of the average currents around the La Hague Cape is to compare the mean sea levels at the different locations. Such a comparison is not easy as different references exist: the marine map reference (z0 h: altitude of the lower astronomical low tide); and the RAF09 French vertical reference to the mean sea level vary widely in this area. Comparisons with the WGS84 Geoid show that z0 h varies from 43.7 to 44.25 m (0.55 m) [30], and RAF09 varies from 47.53 to 47.30 m (0.23 m) between the south-west and north-east of the cape [31]. The larger amplitude of the z0 h variation is explained by its relation to the maximum tidal range that changes a lot in this area.

Table 1 compares the mean sea level measured to the RAF reference, as done by Shom in [26]. We observed that the mean sea level was lower in the middle of the race, at La Roche, Goury and Semaphore, compared with the northern and southern parts of the cape. This difference can be explained by the almost continuous strong currents facing the west and north of the cape, which could result in a mean sea-level variation Δ*z* = *V*^2^/(2 g) (following the Bernoulli's theorem) where Δ*z* is the sea-level variation, g is the gravitational accelerator and *V* the speed of the current. This depression could explain the lowest mean sea-level measurement of 0.13 m at La Roche, 0.40 m below the 0.53 m measured at Port Racine. This would correspond to an average current resulting from the Bernoulli effect of 2.8 m s^−1^. If we account for the difference between La Roche and Herqueville (0.43 m), the corresponding mean speed is 2.9 m s^−1^. These values have the order of magnitude of the average measured and simulated current velocities (figure 7*a*) in the Alderney Race. Bernouilli effects have been already measured elsewhere but principally for instantaneous currents [32–34]. The vertical component of the vorticity as an objective measure of the curvature of the stream lines could also result in local depressions, as shown by Pingree & Maddock [35]. We computed the vorticity field in the Alderney Race. It is significantly close to isolated rocks, but the ambient current is mostly parallel to the shoreline and does not exhibit large sea-level variations at the tidal extension.

We compared the mean sea level measured by our tide gauges and that measured by Shom [26] or simulated with MARS2D. The Shom measurements confirmed the lower mean sea level at Goury, compared with Cherbourg and Diélette (0.31–0.36 m). The differences are slightly lower for the simulation at Goury (0.24–0.27 m), but larger for the semaphore (0.39–0.42 m). Considering possible errors in the terrestrial references for each measurement location and the variation of the RAF reference compared with the geoid in this area, we estimate the error of the mean sea level we measured to be 0.05 m. It could be concluded that a lower mean sea level exists west of the La Hague Cape, which is mainly explained by the average current, as confirmed by the simulation of the calculated average sea level as shown in figure 5*a*. The average lower sea level measured here is exceptional at the scale of the European continental shelf. The simulation (figure 5*b*) shows the locations where lower average sea levels can be found: around the Orkney Islands, mainly in the Pentland Firth, west of the Isle of Anglesey (Irish Sea), St David's Head (Bristol Channel), Ouessant Raz and Barfleur Cape.

Bathymetry maps used by hydrodynamic models are referenced to the observed mean sea level which account with tidal influence. This is an issue because models simulate afterwards the tidal influence. It follows that the models must be set up with a bathymetry map without the influence of tidal currents. This is actually not the case and the bathymetry maps used by hydrodynamic models have an initial sea level lower than the one existing without tide. Only recent bathymetry measurements where the sea bottom is referenced directly to the geoid by accurate GPS measurements can give a homogeneous reference. The resulting error is in the range of 0.1–0.5 m in the Alderney Race, which is exceptional from this point of view (figure 5*b*). Such maps could be applied to correct the bathymetry used by hydrodynamic models.

### (b) Currents

The currents around the La Hague Cape are among the strongest in the world. The pressure gradients generate currents, and the seawater interactions with the bottom and the atmosphere constrain the maximum speeds, direction and the tidal wave propagation.

**Figure 6.**
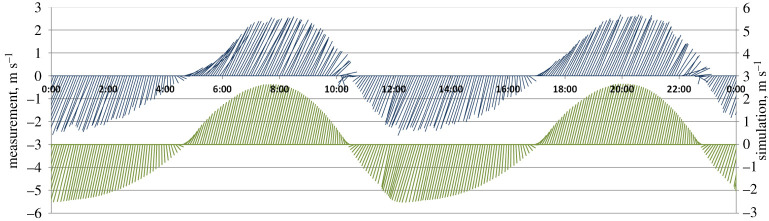
Fishbone diagram of the depth-averaged current vectors measured (top, blue) and simulated (bottom, green) for every 5 min at the station H3, on 27 March 2017. (Online version in colour.)

*In situ* current velocity measurements provide useful local data. However, in the Alderney Race the spatial variability of currents is so large that only a synoptic view can properly describe the existing structures. Simulations by hydrodynamic models provide such a representation once their efficiency has been proven. This validation has been provided by previous studies describing the dispersion of dissolved radionuclide releases from the nuclear plant at La Hague [5,6,10]. Figure 6 displays an example of model/measurement comparison of current velocities at station H3 (figure 2) on 27 March 2017. It shows the model's capacity to reproduce the tidal currents measured at this station. A comparison between measured and simulated current speeds over 41 days with 5 min time steps gives the following relative errors: 1.4% for mean speed with a standard deviation of 14%. Reversing time of the current before low water occurs on average 6 min earlier in simulation with a standard deviation of 22 min. The differences are smaller considering the reversing time before low water, which arrives on average 3 min earlier with a 9 min standard deviation. These discrepancies are probably due to inaccuracies in bottom friction representation at different scales. These differences vary between different locations, in particular those close to the coast.

**Figure 7:**
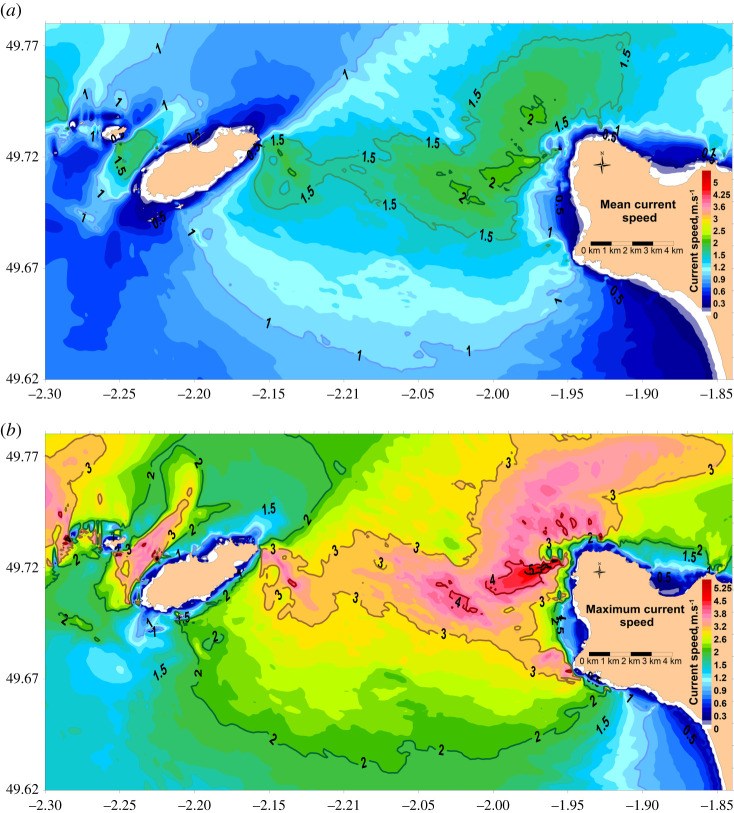
Simulated average (*a*) and maximum (*b*) current speed around the La Hague Cape. The calculations were performed during two moon cycles between 1 March 2015 and 29 April 2015. (Online version in colour.)

Figure 7 shows the simulated average (*a*) and maximum (*b*) currents around the La Hague Cape during two moon cycles, which include a spring tide. The average current confirms the order of magnitude of the mean current facing the locations of the tide gauge measurements (greater than 2 m s^−1^). The distribution is comparable with the average sea level presented in figure 5. This is not the case close to the coast where the calculated mean sea level at −0.3 m between La Roche and Semaphore (figure 5) is associated with a mean current below 1 m s^−1^. The calculated mean sea level accounts for an ‘isolation’ of this area from the locations without offshore currents and corresponds well with the measured mean sea levels (table 1).

The maximum current speed shown in figure 7*b* is a key metric for the design and deployment of tidal converters. This figure also shows where the wind/current interactions are largest.

**Figure 8.**
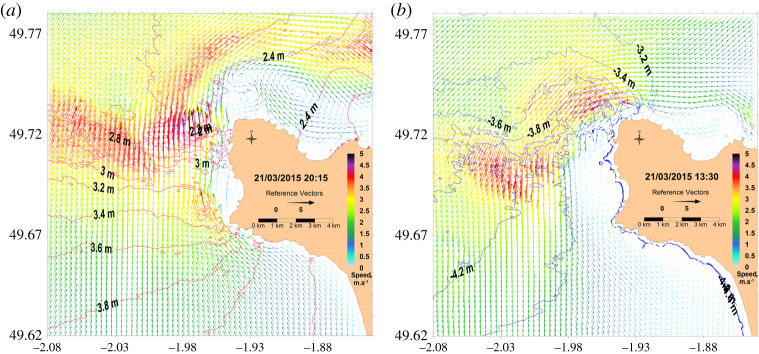
Current velocity vectors (arrows) and sea-level height (contours) at high tide (*a*) and low tide (*b*) on 21 March 2015 (spring tide conditions). (Online version in colour.)

**Figure 9.**
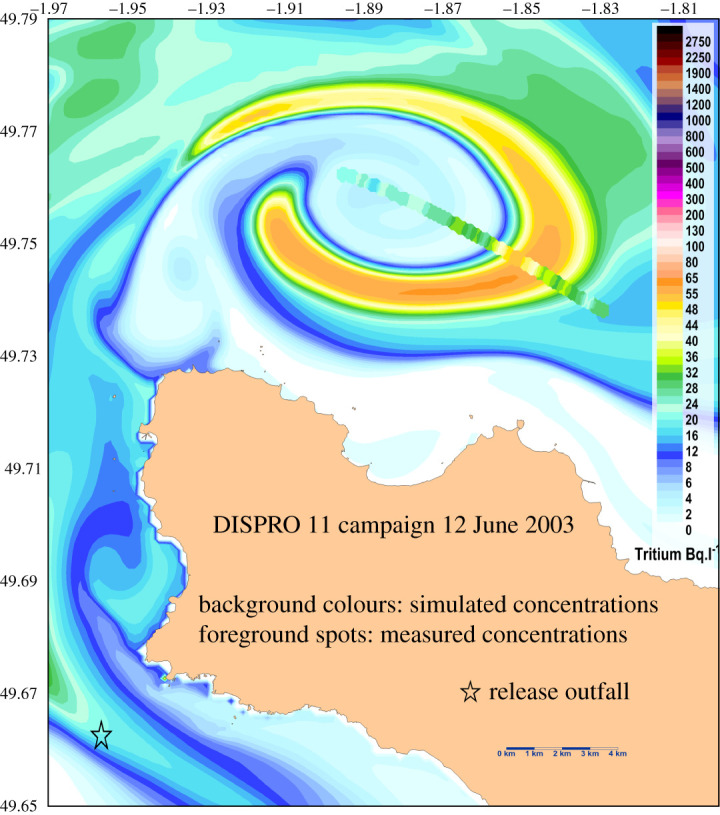
Dispersion of the tritium plume issued from the La Hague release outfall when it reached the north of the La Hague Cape at high tide, simulation (background) and measurement (foreground) [5]. (Online version in colour.)

Figure 8 presents the current vector field for spring tide conditions during the period of peak current at high and low tides. An animation is provided in the electronic supplementary material 1, which details the evolution during two tidal periods. Current speed distributions during high tide and low tide are not symmetrical. During the flood the maximum current speed exceeds 5 m s^−1^ in the north direction and west of the Cape (1°975 W, 49.722N). During the ebb, the maximum current speed reaches 4.5 m s^−1^ in the south-west direction, north-west of the Cape (1°956 W, 49°734N). This results from the asymmetry of the main stream when it arrives in the low-depth rocky plate located north-west from Goury. Another high current speed area is located at 4–5 km, west of the La Hague Cape. It is also influenced by the bathymetry variations, with a larger extent of strong currents from north-west to south-east during high tide. Large sea-level slopes exist during these strong currents, with a difference reaching 2 m at the same time between the south and the north of the La Hague Cape at high tide, and 1.4 m at low tide. At more than 2 km from the coasts, the main stream is relatively the same during the flow and ebb period, except for the progressive generation of a large eddy northward of the La Hague Cape. This eddy has also been evidenced by drifting buoy trajectories [5] and by measurements of labelled releases issued from the outfall of the La Hague reprocessing plant (cf. [5], figure 9).

**Figure 10.**
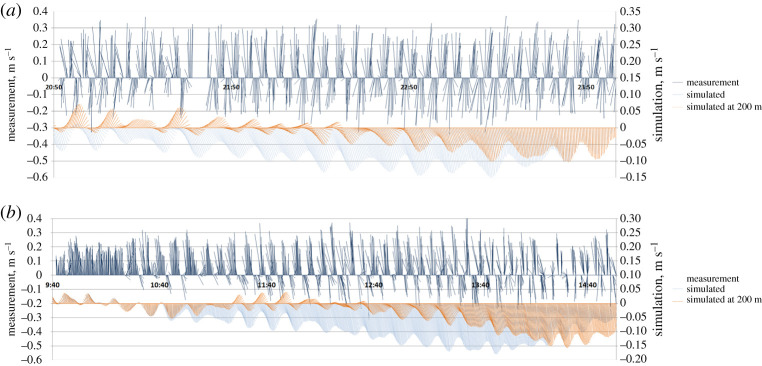
Fishbone diagram of the vertically averaged current vectors measured (top, dark blue) and simulated (bottom, light blue) in the St Martin Bay at station SM. The orange values are simulations at 200 m to the measurement location. (*a*) 2 November 2002; (*b*) 21 November 2002. (Online version in colour.)

**Figure 11.**
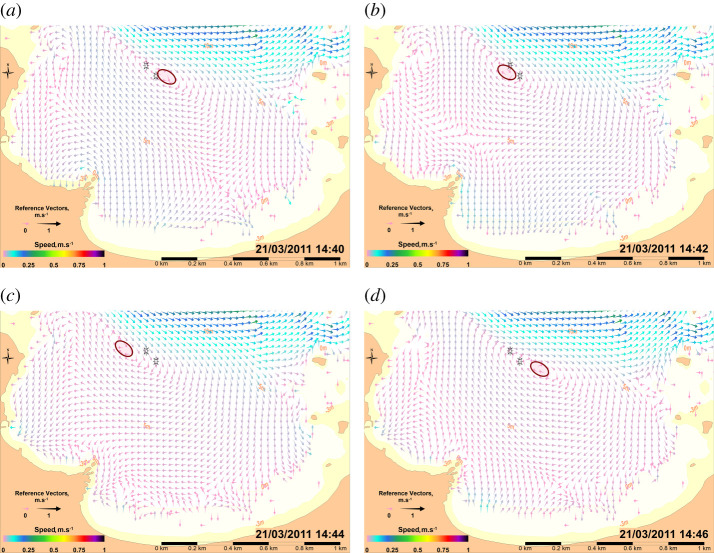
(*a*–*d*) Current vectors in the St Martin Bay on 21 March 2015 every 2 min between 14.40 (*a*) and 14.46 (*d*). The red ellipses show the locations of the centre of the eddy. The black stars show the measurement locations on 2 November 2002 (bottom) and 21 November 2002 (top). The electronic supplementary material 2 shows the whole tidal cycle with a time step of 1 min. (Online version in colour.)

Many eddies and associated counter currents are located along the coasts, behind the capes and in the different bays (St Martin, Ecalgrain and Vauville, figure 2). They are associated with short-term variations due to flow instabilities. The eddies evolve during the tidal cycle and some exhibit short-term periodic variations. Such variations were highlighted during measurements taken in the St Martin Bay (SM station in figure 2, 2 and 21 November 2011). Vertically averaged ADCP measurements obtained every 15 s at station SM over 3 h show a reversal of the current every 5.2 min on 2 November and every 6.6 min on 21 November. The simulation shows variations of the current every 11–12 min on the same dates, with currents twice as low on average compared with measured currents. The differences between measured and simulated currents probably result from the two-dimensional model limits in this context and the precise accounting of bathymetry and bottom friction in this shallow bay (average depth of 10 m). Variations between the centres of the eddy locations explain the inverse current and appear very sensitive. For example, it is largely dependent on the wind influence. Figure 10 shows Fishbone diagrams of these results, together with simulation results obtained at the same point and at 200 m east of the measurement location. Figure 11 explains these variations by the rapid movement of the centre of a gyre from west to east in the St Martin Bay. The rapid change of currents occurs during most of the tidal cycle, as shown by the animation in the electronic supplementary material 2. Similar oscillations can be observed in other coastal areas around the La Hague Cape. This can be interpreted as the effect of the strong current away from the coasts, which generates a resonance effect in the bays and coastal structures. In the St Martin Bay an analogy could be drawn with a police whistle where the air is replaced by seawater. As in a whistle, the gyre in the St Martin Bay is a counter current generated by the strong current occurring north of the St Martin Bay. The magnitude of the current in the gyre is one order weaker than the supposed exciting current. The observed periodic oscillation could be interpreted as a Helmholtz oscillator resulting from ‘a response of a system (the St Martin bay) to an external forcing by developing a restoring force that re-establishes the equilibrium in the system’, as proposed by Rabinovich [36]. In order to calculate the seiche periods in harbours, Rabinovich proposes to apply the following formula for the periods of Eigen (natural) modes in a rectangular basin of uniform depth to calculate the period of the oscillations.

3.1 $$T_{n}\;=\;\;\frac{2L}{n\sqrt{gH}},$$

Where *n* is the number of the mode, *L* is the basin length, *H* is the basin depth and *g* is the gravity acceleration. An application of this method is given in [37]. Parametrizations of this formula are proposed for different basin shapes in [36]. The closest from the St Martin Bay is the semicircular one with *T*_1_ = 2.22 (2*L*(*gh*)^1/2^) for the fundamental mode 1 and *T*_2_ = 0.707 *T*_1_ for mode 2. In the St Martin case, where *L* is around 1000 m and *h* = 10 m, mode 1 gives 7.5 min and mode 2 gives 5.3 min. The value for mode 2 is close to the measured current oscillation (5.2–6.6 min), which reinforces that kind of interpretation. Nevertheless, in the St Martin Bay we measured the oscillation in terms of gyre centre movements instead of sea level.

**Figure 12.**
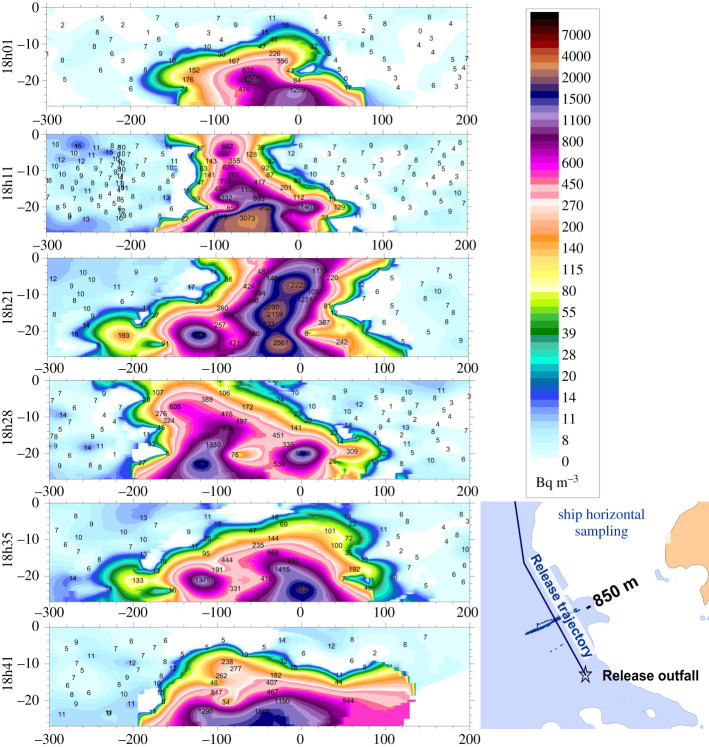
Measured vertical slices of the tritium dispersion during a release from La Hague reprocessing plant at 850 m from the outfall on 8 October 2010. The scale is in metres; the surface is at level 0; the horizontal origin corresponds to the mean release trajectory represented at the bottom right. Each label represents an individual measurement (around 100 for each slice). (Online version in colour.)

If this effect is stronger in the St Martin Bay, simulations show similar instabilities in the Ecalgrain Bay and along the southern coast of the cape (electronic supplementary material 3). Another kind of instability downstream from an isolated rock could also be seen at position −1.923°W, 49.666°N between 18.50 and 22.00 on 21 March 2015. The frequency of this fluctuation increased with the incoming current from the south-east. Another effect of short-term fluctuations could be seen in vertical slices of the tritium-labelled plumes issued from the La Hague outfall that were measured during the DISVER project [6]. In-depth dispersion measurements presented in figure 12 reveal large fluctuations of concentrations at a distance of 850 m downstream to the outfall. Concentration variations occurred in less than 10 min and extended laterally for about 100 m in width. This could have resulted from turbulent fluctuating current or from eddies transported by the current. Realistic simulation of such turbulence effects is still a challenge for marine hydrodynamic models. Theses fluctuations are smoothed at more than 3 km after the release; at this spatial scale the two-dimensional simulation represents the dispersion process well [38].

### (c) Lagrangian currents and dispersion

Dispersion results were obtained from a combination of integrated current and short-scale dispersion processes. They reveal mechanisms from short to large scale such as turbulence, gyres, residual currents and wind effects.

At scales of hours to a few days, instantaneous currents properly explain the variability and complexity of the dispersion measured around the La Hague Cape. A particularity of the Alderney Race that was revealed by scale models and measurements is that it represents a divergence zone for long-term water mass trajectories. This has been explained by Lagrangian residual currents simulated by [19]. This results in different behaviours of the water masses depending on the starting time of a survey or a dissolved tracer. An example of this is given by drifting buoys trajectories obtained in [5]. When dropped at the same initial position, two buoys followed opposite trajectories as described by Lagrangian residual trajectories. In fact, with the same starting position, a different starting time can mean that buoys are launched in water masses distant of up to 30 km. These different water masses could have completely different behaviour in the Alderney Race zone. This particular feature concerns the long-term behaviour of dispersed pollutants. This was confirmed and applied to simulate the dissolved radioactive dispersion [9,10,39].

## 4. Discussion

The results obtained for the Alderney Race and particularly around the La Hague Cape highlight certain particular hydrodynamic processes that are difficult to observe elsewhere. These particular phenomena have not been completely investigated and give way to future studies that could benefit from a significant *in situ* measurement database.

Several questions can be raised, for which the following sections provide some ideas, however, the list is not exhaustive.

### (a) Hydrodynamic model issues

The particularly energetic hydrodynamics of the Alderney Race represents a challenge for environmental hydrodynamic models. They have proven their efficiency in simulating the main processes involved in terms of current, sea-level changes and tracer dispersion. However, many questions remain, particularly in the appraisal of the three-dimensional processes, free surfaces, bottom interaction and turbulence resulting at different scales. Hydrodynamic models are involved in all the aspects mentioned below. If the requirements for models are particularly high, existing data and previous studies give the possibility to strongly constrain the simulation hypotheses and to improve the mechanisms simulated. Results obtained in the Alderney Race could then benefit coastal areas elsewhere.

Considering the numerous mechanisms to take into account in the Alderney Race, hydrodynamic models are essential to interpret *in situ* measurements.

### (b) Bathymetry and bottom friction effects

Bathymetry is the first-order parameter to account for reproducing the hydrodynamics in marine systems. The bathymetry accounted for when establishing hydrodynamic models must not be affected by tidal effects that will be simulated. The bathymetry could then be corrected by the mean sea-level variation resulting from tidal currents. This correction is generally weak in the English Channel (less than 0.1 m). It could be significant in particular locations such as the Alderney Race (more than 0.3 m) and some other locations with strong tidal currents (figure 5*b*). The lower mean sea level measured west of the La Hague Cape (that could be called ‘Goury hole’) must be detailed to confirm differences between the measurements and simulation and the proposed explanation that involves the mean current facing the La Hague Cape.

The observations also provide the opportunity to better account for the bottom friction effect. In fact, in MARS this parameter is represented by the roughness height z0 with the formulation of Soulsby (1995). z0 is a calibration parameter which is set constant everywhere in MARS2D (0.0035 m). This parametrization could definitely be improved by adjusting this parameter with the real space-dependent bottom friction effect. A better representation of this parameter governs the reliability of the simulated hydrodynamics. All differences between measured and simulated currents, mean sea-level fluctuations and tidal range, time phasing and dispersion could potentially be improved, as shown in [40]. The Alderney Race is a sensible place to test z0, which influences the entire hydrodynamic process. The measurements of the tidal wave propagation (table 1) and the spatial variation of the mean sea level could be used to test such parametrization. It influences all the mechanisms presented in the following sections.

### (c) Three-dimensional processes, resonance effects and mesoscale turbulence

To our knowledge the current resonance phenomenon observed in the St Martin Bay has not been measured elsewhere in marine systems (we call it the ‘St Martin whistle’). Simulations confirm that part of the physics of this observation is already accounted for in the MARS2D model. From our different tests, it appears that this resonance is a very sensible phenomenon, in particular with respect to the influence of the wind forcing. Two-dimensional simulations reproduce it with amplitude two times lower and with frequency two times higher, which is probably associated with a harmonic of the resonance frequency. A three-dimensional model would probably better reproduce this effect.

In the Alderney Race, the different scales of gyres and turbulence are influenced by three-dimensional processes, particularly where depth variations are important: along the coast, and offshore tied up to geological features (folds, faults) and trenches around the ‘Fosse de la Hague’ (La Hague Deep). As an example, ADCP profiler measurements show current fluctuations of 1 m s^−1^ occurring within 3 s intervals [14].

### (d) Wind–wave–water mass–bottom interactions

The Alderney Race is a good area to estimate the combined influence of wind–wave–current and sea bottom interactions. As the currents are particularly strong, wind/current interactions are exhausted. The tidal periodic variations and the current rotation around the cape provide many conditions to investigate the wind/current interactions. The generation of waves by local or distant winds could also be studied as done in [40].

Such changing conditions lead to complex situations with measurements that result from the hydrodynamic history at distances upstream of the measurement location. Measurements obtained during the HYD2M project and the development of coupled three-dimensional hydrodynamic models provide ways to account for all phenomena and improve our knowledge of most of the physical mechanisms involved. Recent studies have opened the way [15,40] and those presented in this theme issue have made significant progress. Differences between measurements and simulations show that many questions remain open concerning the turbulence and the kinetic energy transfer at different scales. High frequency and long-term current profiles, at stations or along transects, are powerful tools to understand the mechanisms and check the models. Long-term tide gauge measurements are also sensible means to appraise the wind and pressure atmospheric interactions on water masses at the English Channel scale. In this context, the data acquired during the HYD2M project are particularly interesting with simultaneous records of ADCP [14,40] and radar current profiles, wave measurements and wind vertical profiles.

### (e) Dissolved dispersion

The dissolved dispersion integrates all hydrodynamic processes: mainstream, turbulence, depth variation, diffusion, surface and bottom forcing. The database collected for dissolved radionuclide tracers issued from the La Hague plant enables models to be checked at short to long time scales [11]. This shows that this area is one of the best known for dissolved dispersion.

Recent methods have also been tested that allow for further investigations by applying dye experiments with real-time measurements [41] and that could be applied at the sea surface or in depth for three-dimensional studies.

## 5. Conclusion

It could be concluded from previous and current investigations that the Alderney Race is an exceptional area for coastal hydrodynamic studies. It combines a large range of tidal properties (tidal range, tidal wave propagation) within a restricted area, particularly strong currents exceeding 5 m s^−1^ with associated counter currents and gyres of smaller magnitudes. Variations of depth, coastal topography and orientations provide access to a large variety of original hydrodynamic phenomena. Among them, a 0.4 m drop of mean sea level measured between locations 5 km apart, which appears to be associated with the average currents in the Alderney Race and could be accounted for in order to improve the bathymetry data used by hydrodynamic models applied in this area.

Another particular feature is the observation of a resonance effect linked with the strong offshore currents that generate eddies in coastal areas, particularly in the St Martin Bay. The centre of the local eddy vibrates with a period of 5–7 min. An analogy is made with a police whistle.

Recent studies have exhausted the original processes existing in the Alderney Race, particularly with respect to the future deployment of tidal converters [14,40,42–45]. They could be compared with other potential resources around the UK [46,47]. The available *in situ* measurement datasets are particularly rich with recordings containing: sea level; ADCP current profiles; surface radar currents; waves; dye experiments; surface and in-depth dissolved tracer surveys.

These datasets and available three-dimensional hydrodynamic models enable the complexity of this area to be accounted for and to refine the knowledge of the hydrodynamic process and forcing parameters. The Alderney Race is a natural laboratory to test extreme hydrodynamic conditions. Several future research possibilities have been provided here. Progress in this area could result in a better representation of surface and bottom friction and kinetic energy transfers between the atmosphere and ocean in coastal areas. Applications of these studies could result in a better representation of the tide propagation and tidal currents, and an improvement of hindcast and forecast of dissolved and suspended substances such as pollutants or larvae, or a better representation of sediment transport.

## Data accessibility

Radionuclide measurements are available in: P.B.d.B. IRSN measurements of dissolved radioactivity in seawater, 1982–2016, 39642 stations. Available online: https://doi.pangaea.de/10.1594/PANGAEA.906541 (accessed on 2 October 2019). P.B.d.B. IRSN measurements of dissolved radioactivity in seawater, 1982–2016, including liquid releases database issued from British and French nuclear plants. Available online: https://doi.pangaea.de/10.1594/PANGAEA.906749 (accessed on 2 October 2019). HYD2M project data will be accessible here from 1 July 2020 (station H3 in this work): http://www.geoceano.fr/programmes/anr-hyd2m/ Tide gauge and ADCP data in the St Martin Bay are available upon request to the authors.
